# Beyond Volume: Hospital-Based Healthcare Technology for Better Outcomes in Cerebrovascular Surgical Patients Diagnosed With Ischemic Stroke: A Population-Based Nationwide Cohort Study From 2002 to 2013

**DOI:** 10.1097/MD.0000000000003035

**Published:** 2016-03-18

**Authors:** Jae-Hyun Kim, Eun-Cheol Park, Sang Gyu Lee, Tae-Hyun Lee, Sung-In Jang

**Affiliations:** From the Department of Preventive Medicine and Public Health (J-HK), Ajou University School of Medicine, Suwon; Institute of Health Services Research (J-HK, E-CP, SGL, T-HL, S-IJ), Department of Public Health (S-IJ), Graduate School, and Department of Hospital Management (SGL, T-HL), Graduate School of Public Health, Yonsei University; Department of Preventive Medicine (E-CP), Yonsei University College of Medicine, Seoul, Republic of Korea.

## Abstract

We examined whether the level of hospital-based healthcare technology was related to the 30-day postoperative mortality rates, after adjusting for hospital volume, of ischemic stroke patients who underwent a cerebrovascular surgical procedure.

Using the National Health Insurance Service-Cohort Sample Database, we reviewed records from 2002 to 2013 for data on patients with ischemic stroke who underwent cerebrovascular surgical procedures. Statistical analysis was performed using Cox proportional hazard models to test our hypothesis.

A total of 798 subjects were included in our study. After adjusting for hospital volume of cerebrovascular surgical procedures as well as all for other potential confounders, the hazard ratio (HR) of 30-day mortality in low healthcare technology hospitals as compared to high healthcare technology hospitals was 2.583 (*P* < 0.001). We also found that, although the HR of 30-day mortality in low healthcare technology hospitals with high volume as compared to high healthcare technology hospitals with high volume was the highest (10.014, *P* < 0.0001), cerebrovascular surgical procedure patients treated in low healthcare technology hospitals had the highest 30-day mortality rate, irrespective of hospital volume.

Although results of our study provide scientific evidence for a hospital volume/30-day mortality rate relationship in ischemic stroke patients who underwent cerebrovascular surgical procedures, our results also suggest that the level of hospital-based healthcare technology is associated with mortality rates independent of hospital volume. Given these results, further research into what components of hospital-based healthcare technology significantly impact mortality is warranted.

## INTRODUCTION

Cerebrovascular disease is a major cause of disability and death and is high-risk, requiring safe practices and advanced medical techniques for diagnosis and treatment. Accurate assessment of hospital performance for surgical procedures has become increasingly important since the implementation of pay-for-performance programs designed to link payments to clinical outcomes and public reporting of assessment results.^1,2^ Thus, the success of quality improvement programs, such as pay-for-performance programs, in improving surgical outcomes is based on accurate performance assessments and the ability to identify truly high-performing hospitals.

One of the most simple and easily available performance measures in surgery is the surgical procedure volume of a hospital based on the intuitively attractive “more is better” concept. Considerable evidence exists that higher hospital surgical volume is associated with improved clinical outcomes such as operative mortality,^3^ length of stay, cost,^4^ and survival.^3^

Despite these observations, since the majority of peer-reviewed literature is primarily aimed at determining the presence of the volume–outcome relationships for various procedures, the true mechanism of the volume–outcome association remains in dispute. For example, the magnitude of the volume–outcome relationship varies according to the technical difficulty of the surgery and the availability of specific healthcare technology.^5^ Evidence from qualitative studies^6^ suggests that hospital volume reflects hospital characteristics, such as technical capabilities, personalities of the physicians or staff, culture, leadership, structure, strategy, information, communication pathways, skills training, and physician engagement. These contributors to hospital volume may partially explain the relationship between volume and outcomes. However, they do not fully reveal what volume is a proxy for.

It is also possible that, as prior evidence^7^ has demonstrated, high healthcare technology is associated with lower mortality rates and the improved outcomes are derived from the range of critical care and treatment services offered in high healthcare technology hospitals. Characteristics of high healthcare technology hospitals may also include availability of new technologies, highly equipped operating rooms, better management of health resources, well selected care teams, advanced training programs, multidisciplinary discussions, improved decision making and care, use of standardized management protocols, and appropriate mechanisms to improve treatment that may affect postoperative outcomes.^8–10^

As a result, the use of surgical volume as a meaningful quality indicator and predictor for outcomes in hospital care is questionable. High healthcare technology may be a better surrogate for hospital healthcare quality. A variety of models for measuring hospital-based healthcare technology have been proposed,^11,12^ although the accuracy of these models in predicting outcomes is still unclear. However, practical application of these models is limited because of their complexity. A simple and intuitive method to capture hospital-based healthcare technology and to understand what can serve as a proxy for healthcare quality is desired. Thus, based on recently published novel but simple and intuitive methods^13^ and using current nationwide cohort data from 2002 to 2013, we investigated whether the level of hospital-based healthcare technology was related to 30-day, postoperative mortality rates in ischemic stroke patients who underwent cerebrovascular-related surgery after adjusting for hospital procedure volume.

## METHODS

### Data Sources and Study Design

This study used the National Health Insurance Service-Cohort Sample Data (NHIS-CSD) from 2002 to 2013, which were released by the Korean National Health Insurance Service. Initial cohort members were selected by stratified random sampling using a systematic method to generate a representative sample of the 46,605,433 Korean residents recorded in 2002. Those individuals were followed through 2013. The number of initial cohort members was 1,025,340, approximately 2.2% of the Korean population in 2002.

The healthcare utilization claims data include information on prescription drugs, medical procedures, diagnostic codes based on the International Classification of Diseases, 10th Revision (ICD-10), and healthcare costs. If a member was censored due to death or emigration, a new member was recruited among newborns of the same calendar birth year of the censored member.

We analyzed a unique database of representative individuals who had an ischemic stroke and underwent a cerebrovascular-related surgical procedure. To select our study participants from the NHIS-CSD, we used the ICD-10 code with I63.0 to I63.9 for main diagnosis and further limited the search to include only patients who had a cerebrovascular-related surgical procedure, such as craniotomy for evacuation of a hematoma or craniotomy or craniectomy for decompression. We linked each ischemic stroke patient using a license number to a separate hospital licensure database that included the calendar years. Linking the selected patients to the hospital allowed us to study the association of hospital-based healthcare technology and outcomes during the 12-year follow-up period.

This study was approved by the Institutional Review Board of Yonsei University Graduate School of Public Health (2-1040939-AB-N-01-2016-104).

### Study Variables

#### Independent Variables

For each hospital, the volume of patients undergoing cerebrovascular surgery per year was ranked from low to high using the Rank function provided in SAS software (SAS Institute Inc., Cary, NC; for use in Model 1). We also measured levels of hospital-based healthcare technology implementation based on a range of diagnostic codes for ischemic stroke (ICD-10 codes) recorded over the study period; levels of hospital-based healthcare technology were also ranked from low to high using the SAS Rank function (for use in Model 2). In Models 1 and 2, the volumes of cerebrovascular-related surgical patients and the levels of hospital-based healthcare technology were categorized as low, medium, and high to assess their respective effects on 30-day all-cause mortality. Finally, 9 additional groups (listed in Table 1 ) were devised to assess the combined effects of healthcare technology and volume of cerebrovascular surgeries on 30-day all-cause mortality (Model 3).

**TABLE 1:**
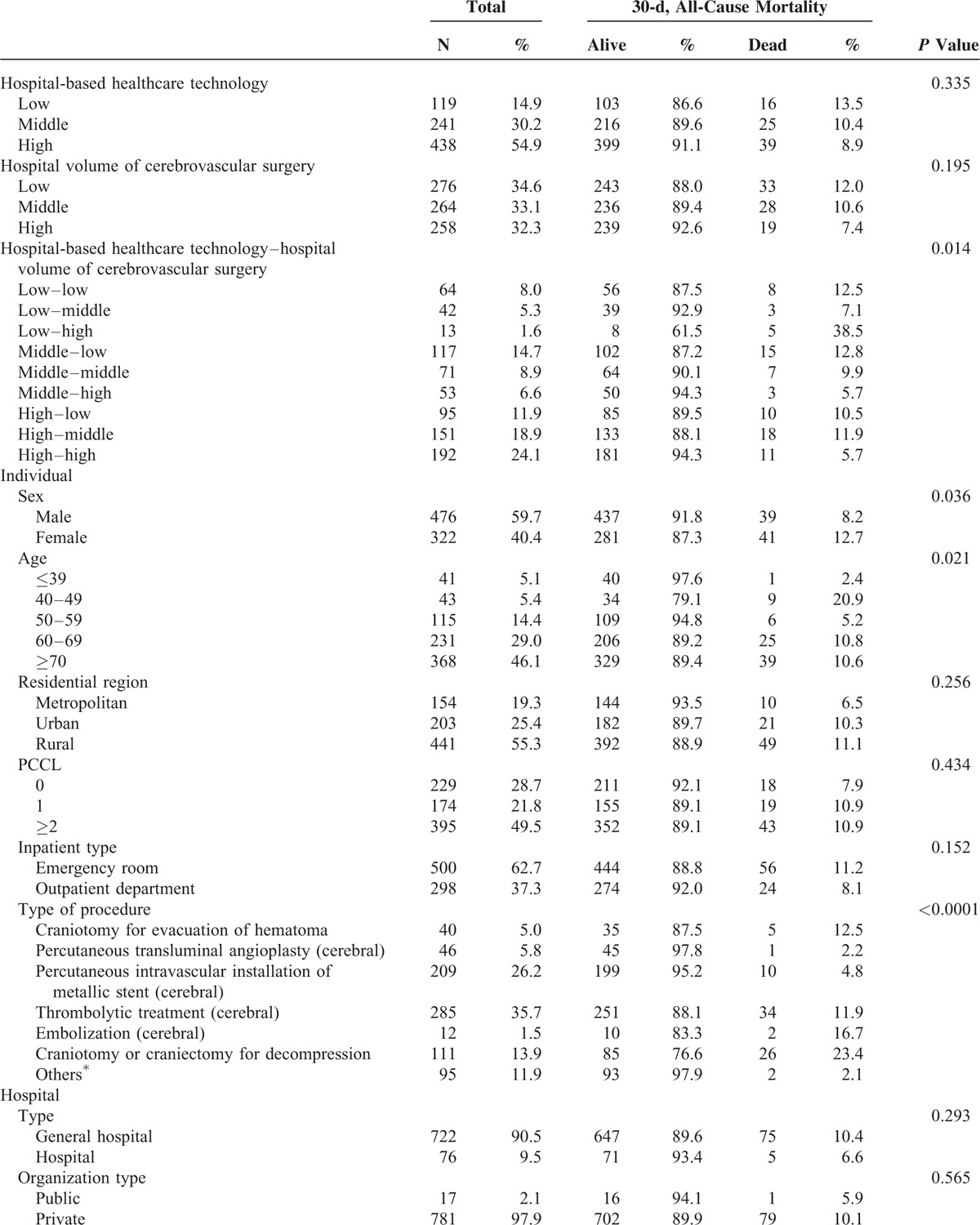
General Baseline Characteristics of Hospitals and Subjects Included in the Analysis

**TABLE 1 (Continued):**
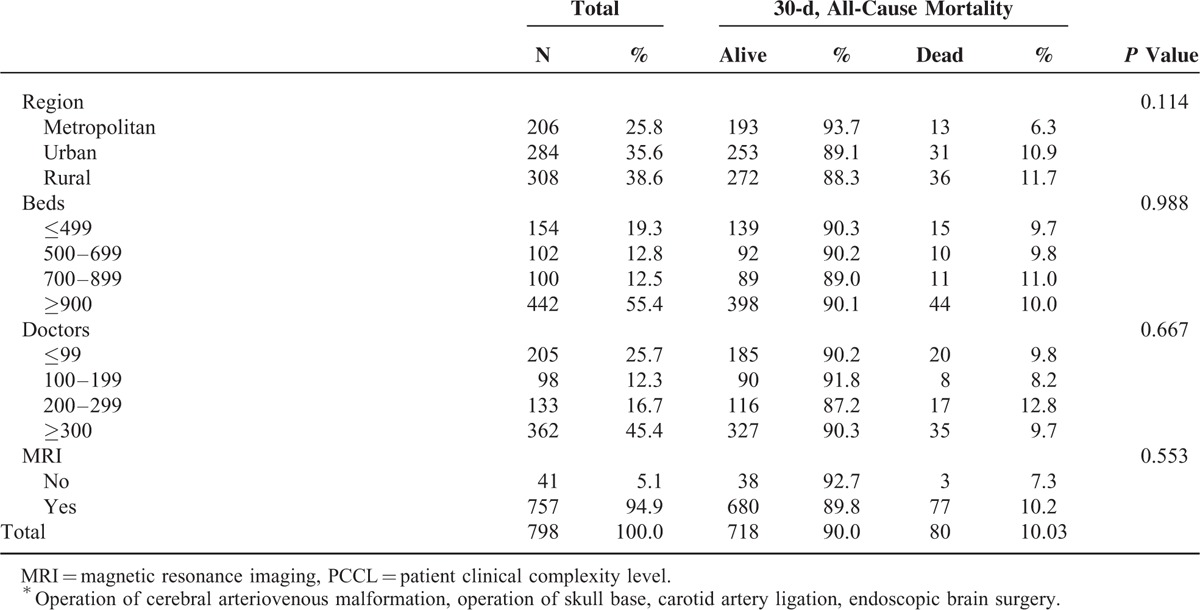
General Baseline Characteristics of Hospitals and Subjects Included in the Analysis

#### Dependent Variables

In this study, the primary end point was 30-day, all-cause mortality after the cerebrovascular-related surgical procedure.

#### Control Variables

Individual characteristics, such as age, sex, and residential region, and hospital characteristics, including hospital type, organization type, region, number of beds, number of doctors, and magnetic resonance imaging (MRI) capabilities, were included as variables that could affect the mortality in the analysis. All covariates were categorical. To adjust for each patient's clinical severity, patient clinical complexity level (PCCL), inpatient type, diagnosis code, and type of procedure were also included in individual characteristics analyzed. Age groups were divided into 5 categories: ≤39, 40 to 49, 50 to 59, 60 to 69, and ≥70. Patient residential regions and hospital regions were categorized as metropolitan (Seoul), urban (Daejeon, Daegu, Busan, Incheon, Kwangju, or Ulsan), or rural (not classified as a metropolitan or urban).

### Statistical Analysis

The primary analysis was based on Cox proportional hazard models. Survival time was defined as the time between 30 days following the cerebrovascular surgical procedure and the date of death. The Kaplan–Meier method was used to plot crude survival curves according to time following cerebrovascular surgical procedure. We plotted a survival curve for mortality and a cumulative curve for 30-day mortality. Considering that critical illness has an effect on outcomes, the Cox proportional hazard model, which is the most frequently used model, might be accurate because it relies on the assumption that the prognostic factors have constant hazard ratios (HRs) over time. For all analyses, the criterion for significance was *P* ≤ 0.05, 2-tailed. All analyses were conducted using the SAS statistical software package, version 9.4 (SAS Institute Inc.).

## RESULTS

### Prevalence of 30-Day, All-Cause Morality

**FIGURE 1:**
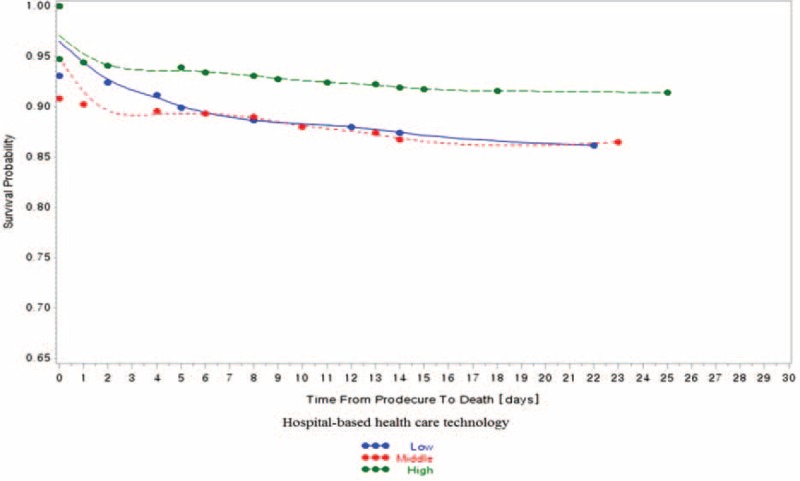
Hospital-based healthcare technology and 30-d, all-cause mortality.

**FIGURE 2:**
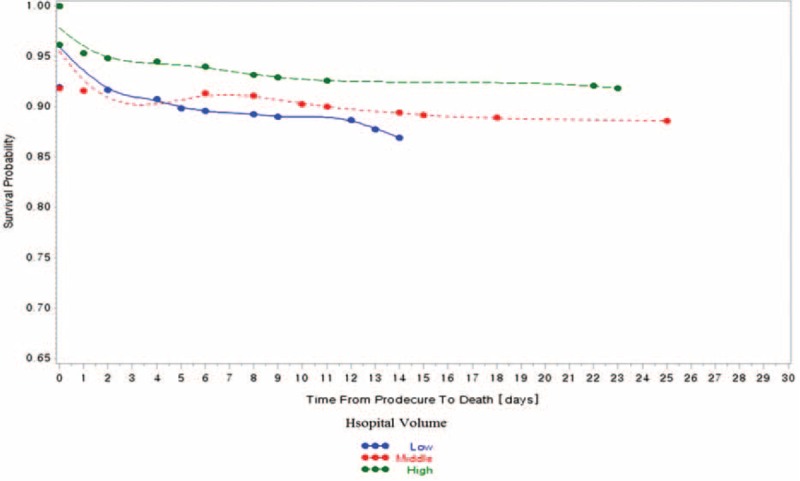
Hospital volume of cerebrovascular surgery and 30-d, all-cause mortality.

**FIGURE 3:**
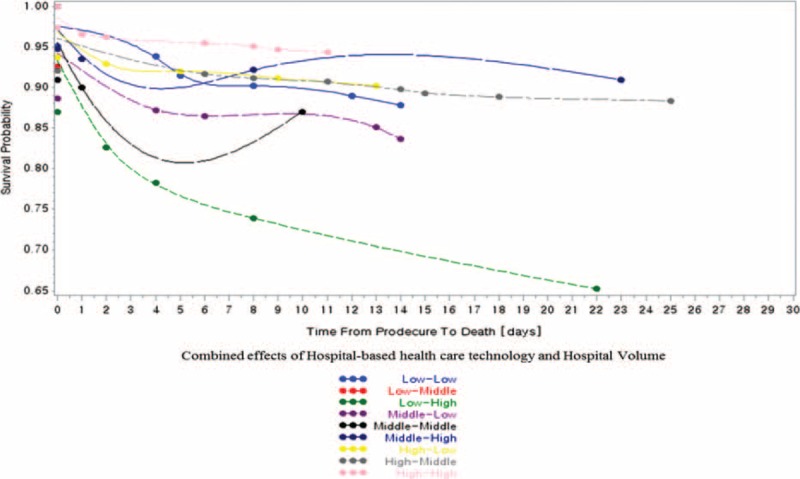
Combined variable analysis between hospital-based healthcare technology and hospital volume of cerebrovascular surgery and 30-d, all-cause mortality.

In the 798 research subjects included in our study, the prevalence of 30-day mortality was 10.03% (80 participants; Table 1 ). Of the total sample, 13.5% of patients with 30-day mortality were treated in low healthcare technology hospitals per year, and 12.0% of patients with 30-day mortality were treated in hospitals with low volume of cerebrovascular surgical procedures per year (Table 1  and Figures 1–3).

### Association Between Hospital-Based Healthcare Technology and 30-Day Mortality

**FIGURE 4:**
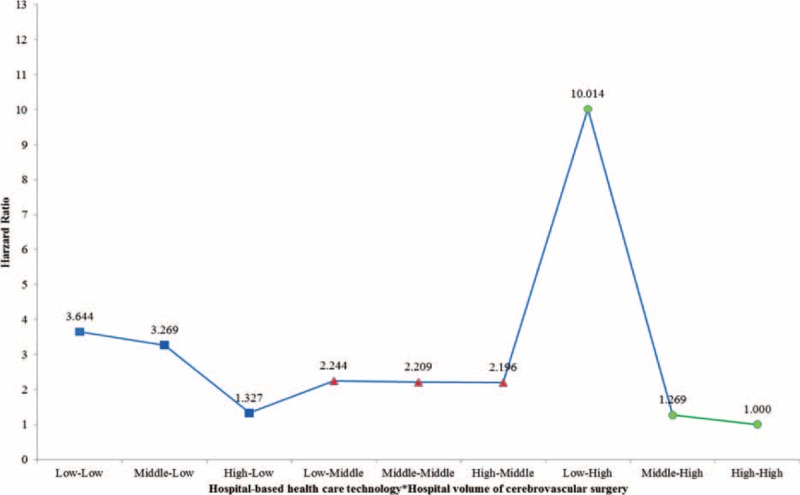
Adjusted association between hospital-based healthcare technology and all-cause mortality (Model 3).

Table 2  shows the risk analysis with adjustments for age, sex, residential region, PCCL, inpatient type, type of surgery, hospital type, organization type, hospital region, number of beds, number of doctors, and MRI capability. After adjusting for all of these confounders, the HR of 30-day mortality in hospitals with low volume (Model 1) was 1.700 (*P* = 0.043) compared to that in hospitals with high volume. After adjusting for hospital volume of cerebrovascular surgical procedures as well as the other confounders, the HR of 30-day mortality in low healthcare technology hospitals (Model 2) was 2.583 (*P* = 0.001) compared to that in hospitals with high healthcare technology. Model 3 examined the combined effects of hospital-based healthcare technology and hospital volume of cerebrovascular surgical procedures as well as the confounders. The HR of 30-day mortality in low healthcare technology hospitals with low volume (low–low) was 3.644 (*P* = 0.004) compared to that in hospitals with high healthcare technology and high volume (high–high; Figure 4). Interestingly, the HR of 30-day mortality in low healthcare technology hospitals with high volume (low–high) as compared to high healthcare technology hospitals with high volume (high–high) was the highest (HR: 10.014, *P* < 0.0001).

**TABLE 2:**
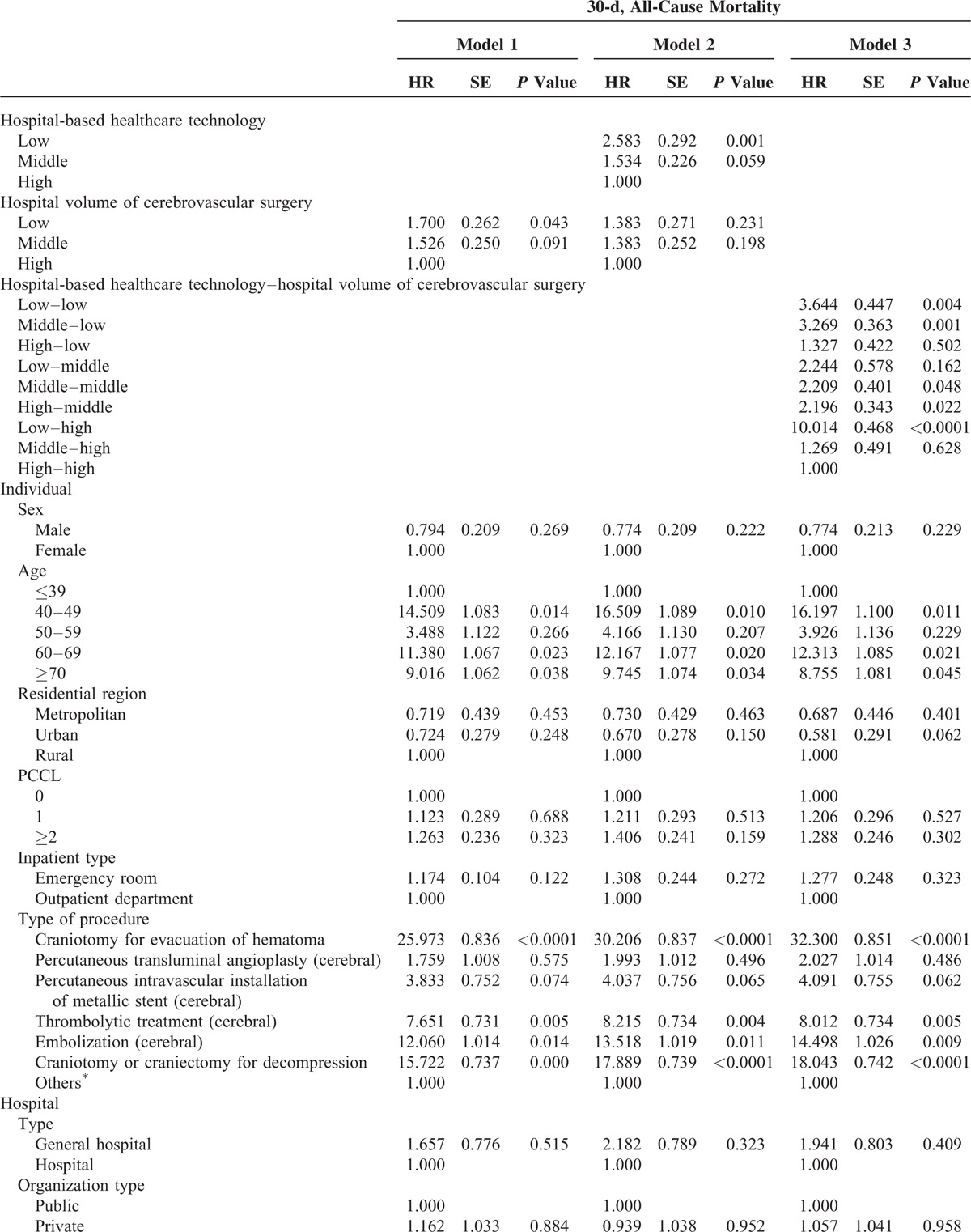
Adjusted Association Between Hospital-Based Healthcare Technology and All-Cause Mortality

**TABLE 2 (Continued):**
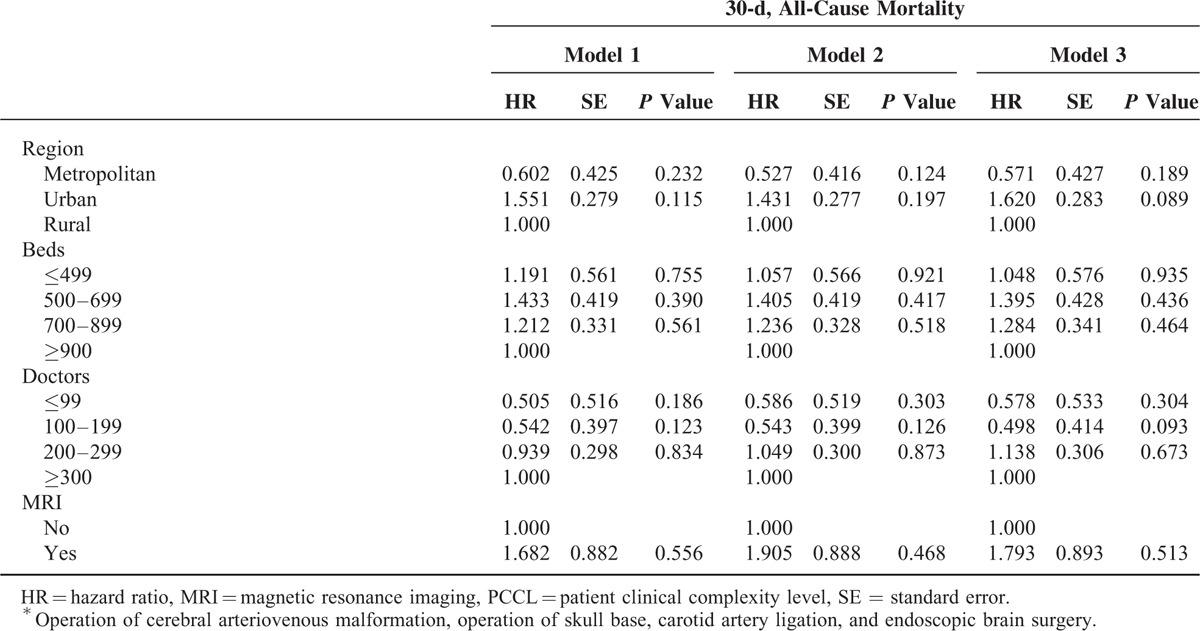
Adjusted Association Between Hospital-Based Healthcare Technology and All-Cause Mortality

Overall, we found that greater implementation of healthcare technology, regardless of the volume of cerebrovascular-related surgical procedures performed, had a marked effect on 30-day mortality rate. The HR of 30-day mortality was the highest for low–high hospitals, while high–high hospitals showed the lowest 30-day mortality rate.

**FIGURE 5:**
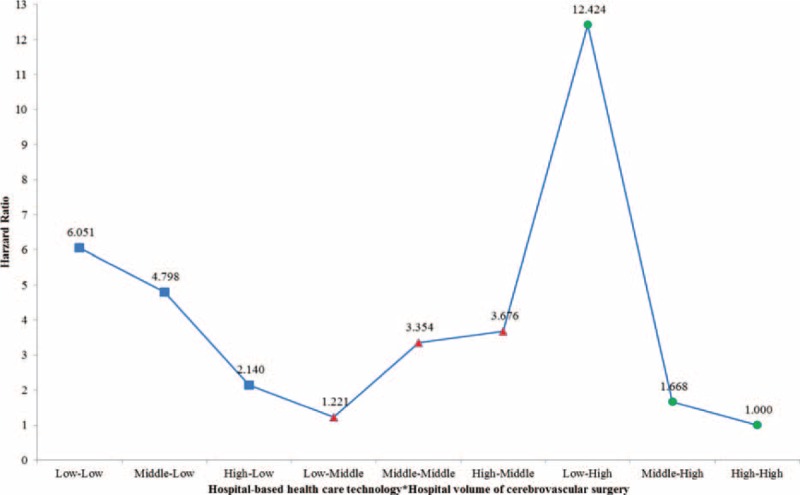
Adjusted association between hospital-based healthcare technology and all-cause mortality in ischemic stroke patients admitted through emergency room (Model 3).

Table 3 comprises results from subgroup analysis of patients who underwent cerebrovascular-related procedures and were admitted through the emergency room, after adjusting for confounders. The subgroups analysis results seemed to show a stronger relationship between hospital-based healthcare technology and volume of cerebrovascular-related surgeries. Nevertheless, the results showed a similar trend comparable to the results for all cerebrovascular-related procedures (Figure 5).

**TABLE 3:**
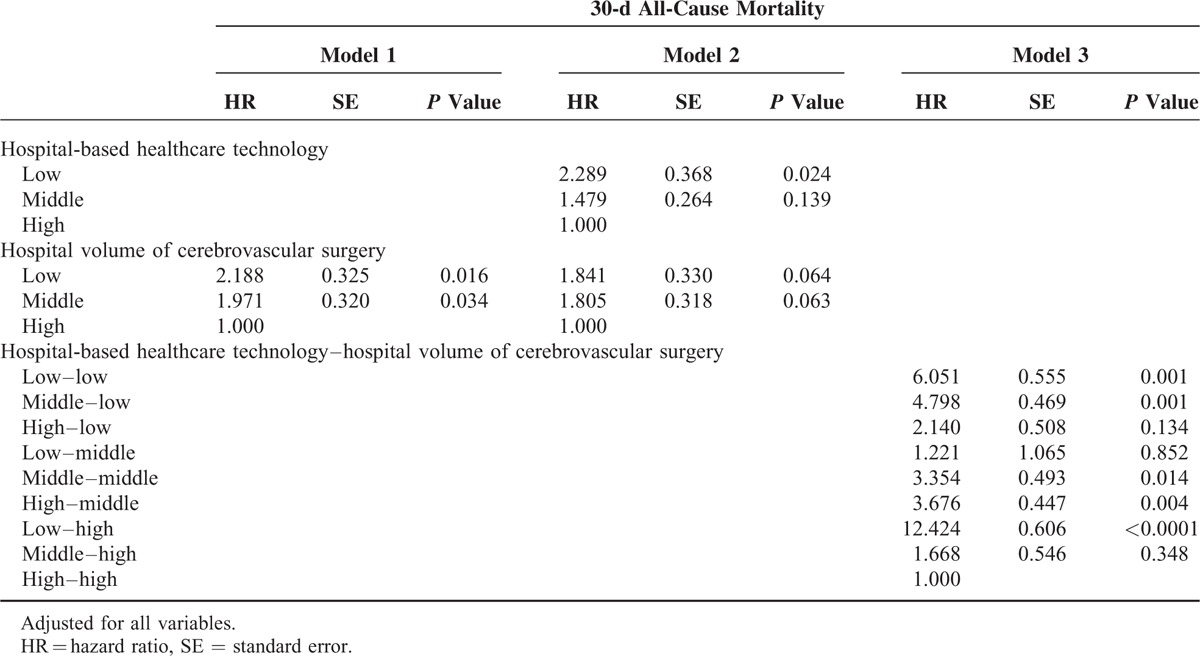
Adjusted Association Between Hospital-Based Healthcare Technology and All-Cause Mortality in Ischemic Stroke Patients Admitted Through Emergency Room

## DISCUSSION

In this study, our primary purpose was to investigate whether hospital-based healthcare technology was responsible for the observed volume–outcome relationship by examining 30-day postoperative mortality rates after adjusting for hospital volume of cerebrovascular-related surgical procedures as well as other covariates in longitudinal models using nationally representative cohort data from 2002 to 2013 in South Korea. The major findings of our study are as follows: hospital-based healthcare technology has a substantial effect on 30-day postoperative mortality among patients diagnosed with ischemic stroke who underwent a surgical procedure (Model 2), although hospital volume of cerebrovascular surgical procedure is also related to 30-day postoperative mortality (Model 1). That is, increased healthcare technology was associated with significantly lower mortality rates, independent of the hospital volume of cerebrovascular surgical procedures. Even after adjusting for the comprehensive array of hospital characteristics such as hospital type, number of beds, number of doctors, etc., mortality rates at hospitals with low healthcare technology were higher than in those with high healthcare technology.

We also found that patients diagnosed with ischemic stroke treated in hospitals with low healthcare technology, regardless of the hospital volume of cerebrovascular surgical procedures, had the highest 30-day mortality rate, followed by patients diagnosed with ischemic stroke treated at middle healthcare technology hospitals. Patients diagnosed with ischemic stroke treated at high healthcare technology hospitals had the lowest 30-day mortality rate, regardless of the hospital volume of cerebrovascular surgical procedures; but the 30-day, all-cause mortality rate in hospitals with low healthcare technology and high hospital volume (low–high) was the highest.

The results of our study provide very insightful scientific evidence into the association between hospital-based healthcare technology and 30-day mortality following complex surgical procedures. In this study, we addressed the notion that volume is a predictor of mortality by showing the independent effect of hospital-based healthcare technology, beyond hospital volume, on mortality.

Although hospital-based healthcare technology level provided a straightforward predictor for outcome, 1 argument against its use may be that hospital-based healthcare technology is simply a proxy for size or volume. In fact, hospital-based healthcare technology used for identifying these mechanisms was correlated with size or volume. As in a previous study,^13^ our study showed that approximately 40% of the 539 hospitals in the highest healthcare technology quintile were medium- or smaller-sized hospitals; however, our results were far from identical to those in the other study.^13^ In addition, the hospital volume-to-outcomes relationship remains controversial.^14,15^ Luft et al^16^ examined 2 hypotheses in their study. The first “selective referral” hypothesis indicates possible reverse causality in the volume–outcome relationship: physicians and hospitals with improved patient outcomes attract higher volumes of patients. The second “practice-makes-perfect” hypothesis is based on the mechanism of “learning by doing,” by which providers achieve improved patient outcomes as a result of increased expertise resulting from the increased volume. However, Huesch^17^ and Tsai et al^18^ concluded that learning and selective referral effects played no significant role in the volume–outcome relationship. That is, volume alone does not result in improved patient outcomes.^19,20^ In addition, it remains unclear whether volume always leads to improved clinical judgment, better patient selection, or reduced technical errors. Thus, there is considerable consensus that volume is an imperfect correlate of healthcare quality given that availability of new technologies and better management of health resources, multidisciplinary discussions, etc., have improved considerably.

It is also clear that, as hospitals or surgeons perform cerebrovascular surgical procedures with high levels of healthcare technology, they can reduce their patients’ mortality rates. It is also possible that these high levels of healthcare technologies may relate to the well selected teams of healthcare providers with advanced training programs, including nursing staff who are brought together by specialty-trained surgeons. These highly trained providers can implement standardized clinical pathways, protocols, and appropriate mechanisms to provide proper treatment that might improve the safety of cerebrovascular surgical procedures.

Therefore, although a hospital volume–mortality relationship in cerebrovascular surgical procedures existed in our study as in previous studies,^21–26^ this study serves as a reminder that hospital-based healthcare technology is an important explanatory predictor underlying the relationship between hospital volume and mortality. Thus, further research is necessary to identify the key processes of quality of care in hospitals performing cerebrovascular surgical procedures that impact mortality. Such processes may include availability of sophisticated services and quality improvement program such as clinical pathways and protocols.

In addition, although high surgical techniques, and care processes are essential for improving the care and outcomes of surgical patients, the cost for developing and utilizing new medical technologies is high.^27^ Actually, the United States spent 2.9 trillion dollars on health care in 2013.^27^ This figure continues to rise and has been a significant economic burden with legislative efforts aimed at flattening the cost curve. However, this is not necessarily bad as long as the new technologies result in clear outcome improvements. In addition, health outcomes/the costs defined as value^28^ are difficult to actually measure when applied to surgical care and technologies in healthcare domains due to the fact that its elements are dynamic. Improved outcomes associated with changes in cost of technology driven by competition and changes in market share alter the equation over time.^29^ Therefore, further study will also be essential in identifying the clinical domains in which healthcare technology is most beneficial, as well as investigating if there is a relationship between healthcare technology and cost of care.

Our study has a number of strengths and limitations. The participants in the survey are representative of the overall South Korean cerebrovascular inpatient population. Our large and longitudinal cohort sample size allowed the results to be generalized to the adult South Korean population. Nevertheless, several limitations that may have affected our results need to be considered in the interpretation of our findings. First, when we selected participants for our study, both ICD coding and cerebrovascular surgical patient characteristics were considered. However, because the categorization of hospital-based healthcare technology relied on ICD coding of principal diagnosis, it is difficult to validate individual ICD codes. Our data are from a deidentified database, making it susceptible to errors related to coding. Second, as this is a large and longitudinal nationwide sample, there may be significant heterogeneity in the care provided both in the field and at receiving hospitals. Thus, we cannot comment on which aspects of patient care most affected survival. Third, although unmeasured hospital characteristics, such as the availability and quality of protocols used, may contribute to outcomes, we could not analyze the contribution of these hospital characteristics because of the limited information in the claims database. Finally, in this study, although specialty hospitals dedicated to a narrow range of procedures, but may in fact be institutions with high healthcare technology, we could not excluded specialty hospitals due to lack of information. Nevertheless, although this limits the generalizability of our study, the majority of participants still take place in general hospitals.

## CONCLUSION

This study provides scientific evidence suggesting that hospital-based healthcare technology is the most important variable underlying the relationship between hospital volume and mortality. Volume alone is an imperfect correlate for quality. Higher levels of hospital-based healthcare technology have a strong association, independent of hospital volume, with decreased mortality. Therefore, further research to identify the key components of technology that affect mortality is warranted because the evidence in this area is lacking.
