# Beyond Volume: Hospital-Based Healthcare Technology for Better Outcomes in Cerebrovascular Surgical Patients Diagnosed With Ischemic Stroke: A Population-Based Nationwide Cohort Study From 2002 to 2013: Erratum

**DOI:** 10.1097/01.md.0000484042.43613.b3

**Published:** 2016-05-20

In the article “Beyond Volume: Hospital-Based Healthcare Technology for Better Outcomes in Cerebrovascular Surgical Patients Diagnosed With Ischemic Stroke: A Population-Based Nationwide Cohort Study From 2002 to 2013”,^1^ which appeared in Volume 95, Issue 11 of *Medicine*, Dr. Tae-Hyun Kim's name was originally misprinted as Tae-Hyun Lee. The article has since been corrected online.
